# Constructing Pseudowords with Constraints on Morphological Features - Application for Polish Pseudonouns and Pseudoverbs

**DOI:** 10.1007/s10936-022-09884-6

**Published:** 2022-08-05

**Authors:** Joanna Daria Dołżycka, Jan Nikadon, Magdalena Formanowicz

**Affiliations:** 1 Nicolaus Copernicus University, Toruń, Poland; 2 Department of Applied Emotion and Motivation Psychology, Ulm University, Ulm, Germany; 3 SWPS University of Social Sciences and Humanities, Warsaw, Poland; 4 Institut für Psychologie und Pädagogik, Abteilung Angewandte Emotions- und Motivationspsychologie, Albert-Einstein-Allee 47, D-89081 Ulm, Germany

**Keywords:** Pseudowords, Grammar, Linguistic processing, Psycholinguistics, Wuggy

## Abstract

Pseudowords allow researchers to investigate multiple grammatical or syntactic aspects of language processing. In order to serve that purpose, pseudoword stimuli need to preserve certain properties of real language. We provide a Python-based pipeline for the generation of pseudoword stimuli that sound/read naturally in a given language. The pseudowords are designed to resemble real words and clearly indicate their grammatical class for languages that use specific suffixes from parts of speech. We also provide two sets of pseudonouns and pseudoverbs in Polish that are outcomes of the applied pipeline. The sets are equipped with psycholinguistically relevant properties of words, such as orthographic Levenshtein distance 20. We also performed two studies (overall *N* = 640) to test the validity of the algorithmically constructed stimuli in a human sample. Thus, we present stimuli that were deprived of direct meaning yet are clearly classifiable as grammatical categories while being orthographically and phonologically plausible.

## Introduction

Paradoxically, when investigating natural language processing, researchers are often compelled to employ pseudowords and artificial languages. Pseudowords are used in psycho- and neurolinguistic studies because they allow researchers to abstract from the actual meaning and context conveyed by real words. The use of pseudowords enables better control of the morphological (Longtin & Meunier, 2005; Snyder, 1995), semantic (Dorffner & Harris, 1997)⁠ and syntactic (Opitz, 2004)⁠ properties of stimuli, which in turn allows researchers to disentangle the specific role of these features in language processing.

Yet, to ensure that pseudowords can be used in a meaningful way, they must be devised with care and control. In this paper, we describe former methods of pseudowords generation and point out their advantages and disadvantages. As a following step, we propose a novel procedure of pseudoword generation based on the Wuggy algorithm. We augmented the basic procedure with language-specific rules based on linguistic priors and real language-use data, which allows for the distinction of grammatical categories. As an example of application of this procedure, we provide a Python-based script for (Polish) pseudoverb and pseudonoun construction. Additionally, we provide two grammatically diverse sets of stimuli in Polish (i.e., pseudonouns and pseudoverbs with comparable psycholinguistically relevant properties) that can be used for various research applications. The first set contains 159 pseudowords with a variety of stems and suffixes typical for a specific grammatical class (noun or verb). The second set (242 pseudowords) contains pseudonoun-pseudoverb pairs that share the common stems. These two example pseudoword stimuli sets can be used in psycholinguistic research that focuses on the differences in processing words that belong to the mentioned grammatical classes, especially by those contributing to the ongoing debate on the differences in the processing of nouns and verbs (Vigliocco et al., 2011). In this article, we focus on verbs and nouns as an example. Importantly, however, the proposed method could be easily extended to any grammatical category, and thus, it offers a broader framework of pseudoword construction whenever some specific morphological features of pseudowords are of special consideration. Finally, we provide an open-access Python-script that implements our pseudoword generation procedure and can be used by other researchers to generate similar datasets in other languages or to impose other morphological constraints on the pseudowords generated.

## Psycholinguistically Relevant Properties of Pseudoword Stimuli

Examining word processing mechanisms by employing pseudowords is an established methodological approach (Heim et al., 2007; Kissler & Herbert 2013; Price et al., 1996). A particular advantage of using pseudowords in linguistic research is that they are devoid of literal meaning, which allows for the evaluation of nonsemantic aspects of language processing. Pseudowords also facilitate control over a multitude of other potentially confounding features of linguistic stimuli (that are difficult to manipulate, manage, and account for in case of real words), for example, imageability (Tyler et al., 2002) and age of acquisition (Kuperman et al., 2012). The advantages of pseudowords are appreciated in many contexts. First, in investigation of sublexical processing and its relationship to cognitive function, pseudowords are used as stimuli to test mechanisms of cognitive models of reading (Coltheart et al., 2001; Grainger & Jacobs, 1996; Harm & Seidenberg, 2004). Second, pseudowords are useful when social aspects of language are studied. Presenting vocal stimuli that are detached from meaning but contain frequency and intensity cues corresponding to real emotional words allows for examining the impact of prosodic factors in linguistically induced emotion processing (Kissler & Herbert, 2013; Preti et al., 2016). Also, an important application of pseudowords can be found in examinations of grammar—and specifically whether grammatical categories deprived of literal semantic content have the metasemantic effect of conveying meaning through grammatical cues (Formanowicz et al., 2017). However, due to structural differences among languages, preparing stimuli that reflect universal rules for processing grammatical classes can be very difficult if not impossible (e.g., Vigliocco et al., 2011).

When constructing pseudowords for various research applications, however, some features need to be taken into account. First, pseudowords should be constructed with respect to the statistical properties of real word morphology. In that sense, pseudowords differ from nonwords with respect to orthographic and phonological regularities (Rosinski & Wheeler, 1972). According to Ziegler et al., (1997), in perceptual identification tasks, words and pseudowords show reaction-time-related priority effects over nonwords. This means that, if the presented stimulus is more word-like (complies with linguistic rules of a given language), the reaction time for its identification as a language-related item will be shorter. This indicates that, in many research situations, it is not enough to use nonwords (random letter strings), but stimuli must be constructed according to certain rules in order to be treated as linguistic stimuli regarding the amount of word-specific information that they carry. Barca and Pezullo’s (2012) study supports this conclusion. They presented three types of stimuli—words, pseudowords, and random letter strings—and participants were asked to classify stimuli as linguistic or nonlinguistic. The results of a computer mouse movement trajectory analysis demonstrated the presence of a lexical dimension line, which is a continuous measure of recognition of linguistic stimuli. Real high- and low-frequency words were stable and correctly recognized as lexical stimuli, pseudowords were problematic but eventually categorized as nonlexical stimuli, and letter strings were clearly judged as nonlexical stimuli. These findings demonstrate that nonwords and pseudowords are processed differently and the latter need to meet specific linguistic criteria that should be rigorously controlled to be considered a linguistic unit.

Second, when designing pseudoword stimuli, not only should the resemblance to real words be considered, but also the frequency of real word occurrences from which pseudowords originate. A study by Perea et al., (2005) found that pseudowords generated from high-frequency real words yielded less reaction-time latency than pseudowords generated from low-frequency real words because the verification mechanism of the high-frequency base of pseudowords facilitates its comprehension.

The third important feature in pseudoword construction is the frequency of the first syllable (Chetail & Mathey, 2009). According to Carreiras et al., (2006), behavioral and neural responses can differ depending on the first-syllable frequency. Thus, from the linguistic research perspective, the probability of obtaining phonetically correct syllables (N-grams) is another important factor of pseudoword construction. The first-syllable frequency impacts word naming and lexical decision tasks, where multisyllabic words with frequent first syllables are named more quickly than those with less frequent ones, but they elicit slower reaction times in decision tasks (Álvarez et al., 2000). Thus, pseudoword construction should also consider the first-syllable effect to enhance the efficiency of pseudoword reading fluency.

The fourth feature regarding the construction of pseudowords is their similarity to real words. Neurolinguistic research has shown that pseudowords that are more similar to real words can elicit faster (and, in a neurophysiological sense, stronger) responses than stimuli that are more distant from real words (Dorffner & Harris, 1997)⁠. Thus, the similarity between pseudowords and real words should be controlled. Typically, pseudowords used in research should be generated from high-frequency real words, but they also should not be too similar to real words to avoid evoking unintended associations with real word semantic content and an overlapping of the neural activity associated with this content. Real words are typically considered to be similar in three different ways (Siew, 2018): semantically (i.e., with respect to their meaning, e.g., *nail*, *hammer*, and *mallet*), phonologically (i.e., with respect to their sound in spoken language, e.g., *chute* and s*hoot*), and finally, orthographically (i.e., with respect to their spelling in written language, e.g., *nail* and *mail*). In the case of languages that use nonlogographic writing systems (e.g., alphabetical), phonological and orthographic similarity are strongly associated. In the case of pseudoword-based auditory stimuli, their phonological similarity to real words is of greater importance, while for pseudoword-based visual stimuli, their orthographic similarity to real words should be of primary consideration. For example, orthographic similarity affects the speed of recognition of pseudowords; a high number of orthographic neighbors leads to faster responses (Huntsman & Lima, 2002).

The extent of the orthographic neighborhood of a (pseudo)word is most often quantified using either the *Coltheart’s orthographic neighborhood size* metric (ON, *Coltheart’s N*) or *orthographic Levenshtein distance 20* (OLD20; Yarkoni et al., 2008). ON can only be applied to strings with the same number of characters and is a strictly binary-based measure (two strings are considered to be neighbors if one of them can be transformed into the other by a single letter substitution, e.g., *brain* ↔ *train*). This approach to orthographic similarity quantification is considered relatively restrictive (Yarkoni et al., 2008) because the perceptual similarity of words (or other character sequences) can also be achieved by letter insertion (e.g., *widow* → *window*), deletion (e.g., *planet* → *plane*), or transposition (e.g., *trail* → *trial*), none of which is considered when using the ON approach (Yarkoni et al., 2008). The OLD20 method overcomes these constraints, as it is based on Levenshtein distance (LD; Levenshtein 1966), a string distance metric widely used in computer science. The LD between two words is defined as the minimum number of substitutions, insertions, or deletions required to transform one string into the other. Thus, it quantifies the orthographic distance between two character sequences in a nonbinary manner, and moreover, it is not limited to the comparison of strings of the same length. For example, for the words *brains* and *transit*, we get LD = 4 because the four possible operations that can generate the second word from the first are (1) substitute “b” for “t” → *trains*; (2) delete “i” → *trans*; (3) insert “i” → *transi*; and (4) insert “t” → *transit*. Yarkoni et al., (2008) defined OLD20 as the mean LD from a given word (in this research, a pseudoword) to its 20 closest LD-based orthographic neighbors (in this research, real words). This implies that the calculation of OLD20 for a given character sequence requires the computation of LD between this sequence and every word in a given (reference) lexicon before the selection of the top 20 of its closest neighbors can be made and their average LD distance can be calculated. Due to these advantages of OLD20 over the ON measure, in this research we relied on the former to quantify the orthographic distance between the pseudowords produced and real words.

Finally, when constructing stimuli for psycholinguistic and neurolinguistic studies, one must consider that each language has unique properties, and it is impossible to create universal stimuli sets that can be applied to all or many different languages. For example, Polish and English have significantly different structural properties. In terms of linguistic typology, Polish is an inflectional language (Polański, 1999); the grammatical function of a word is determined by its inflectional ending, which communicates information about the word’s grammatical class (e.g., verb, noun, or adjective) and inherently conveys a partial meaning of the word, for example, by including a grammatical gender exponent in nouns or adjectives. In contrast, in English, which is an isolating (analytical) language with a fixed sentence pattern (SVO), the grammatical function of words within a sentence is conveyed by word order (Moravicsik, 2013). In other words, the easiest way to determine the grammatical class of a word is through the sentence pattern (e.g., *I desert the desert*, where the grammatical class of the word *desert* in its written form could not be grammatically distinguished without the context). In Polish, and other inflectional languages, due to the variety of inflectional exponents, it is easier to distinguish individual lexical forms, regardless of their order in the sentence. That is to say, grammatical classes can be recognized by looking at the word itself (e.g., *bieg-ø* is a noun, while *bieg-a-ć* is an infinitive verb form; in contrast, *run*, the English translation, can only be disambiguated by adding “the” or “to”). One method for resolving differences in grammatical class processing is to employ pseudowords with suffixes that clearly indicate a particular grammatical class, which is possible in Polish and other inflectional languages.

The approach presented in this article provides a solution to the problem of homonymy of linguistic units encountered in the single word presentation paradigm. When words have the same forms (e.g., *run*), they are grammatically indistinguishable without an auxiliary word (“to” or “the”) as in positional languages. Our method results in the presentation of a pseudoword as a single letter string with a fixed complexity (pseudoword stem and grammatical exponent) rather than a stimulus consisting of two separate letter strings. Such pseudowords are best suited for researchers who want to investigate the cognitive connotations associated with understanding concepts embedded in grammatical classes. We focus here on pseudoverbs and pseudonouns, to refer to the current debate addressing the issue of whether syntactic or semantic features of words have an impact on differences in functional processing in psycholinguistic tasks (Vigliocco et al., 2011). Given the importance of studying verbs and nouns as main cognitive concepts and sentence-building grammatical categories, we propose a novel method of constructing pseudoverbs and pseudonouns that meets the highest standards of psycholinguistic research. Interestingly, in pseudoword construction paradigms, there is no method that preserves grammatical class while still maintaining control over the psycholinguistic features of a word. Before we introduce such a method, we will first introduce other commonly used methods of pseudoword generation.

## Methods of Pseudoword Generation

One of the first widely used methods of non- and pseudoword production was letter substitution. Basically, the letter substitution method is based on random letter selection and the substitution of those with other letters. This results in obtaining one or more pseudowords from one real word. For example, a pseudoword like *rompuner*, *comnumer*, or *cormuter* could be obtained from the word *computer* by substituting one or two letters (Brown et al., 1987). For example, this method was used to generate a list of 3,023 Polish nouns for linguistic research (Imbir et al., 2015) and in the psychology of language field within studies on the verb–agency link (Formanowicz et al., 2017). It was also used in the English Lexicon Project (Balota et al., 2007), in French (Ferrand et al., 2010), and in Dutch (Keuleers et al., 2010). Although widely used, this method of generating pseudowords does not guarantee that the produced items will meet the linguistic criteria due to the lack of simultaneous evaluation of features, such as the frequency of bigrams and trigrams. Therefore, pseudowords are often also evaluated by competent judges who determine whether a given string of letters can be considered a pseudoword or not in order to select units that are subjectively felt to resemble real words but lack literal meaning. The judging criteria, however, are chosen subjectively by the researchers. For example, some pseudoword evaluation criteria are based on whether pseudowords are constructed from existing or potential syllables or if there is a possibility that the given pseudowords may be read fluently (i.e., if they are pronounceable; Imbir et al., 2015). This is often insufficient, because it is possible to fluently read a pseudoword beginning with the letter ę (*ęgatek*) or the consonant cluster “pf” (*pfkiczyć*), yet these pseudowords do not follow the phonotactic and orthographic rules of Polish language (none of the real Polish words begin with those letters). Additionally, it is possible that the syllables of pseudowords ending in vowels could be considered probable due to the open syllable rule (a phonetic rule of the Proto-Slavic language from which the Polish language originated), whereby each syllable in a word most often ends with a vowel. Given this, words that have syllables ending in a vowel may sound familiar to natural language users, leading to an expansion of the criteria for including pseudowords (for example, ignoring the rules described above). The lack of algorithmic control over stimulus production increases the frequency of atypical occurrences of phonetic clusters within the syllable. Overall, there are a number of factors that can affect stimuli created with the use of the mentioned method, thus potentially inducing a bias in the results of studies on how people process linguistic items.

Over time, methods of generating pseudoword items have become more technically advanced. Researchers have developed programs, such as MCWord (Medler & Binder, 2005), WordGen (Duyck et al., 2004), and WordCreator (Trost, 2002), to produce pseudowords. All these programs work at the letter level and perform N-gram matching using language rules defined in the software settings. Such methods are based on an amalgamation of subword units (typically syllables) to obtain pseudowords that meet the eligibility criteria for the chosen language. The most important criterion is based on the observation that in real, natural language some bigrams and trigrams have higher frequency than others (Suen, 1979). Thus, their overall frequency distribution should be preserved in the pseudowords produced (e.g., Solso et al., 1979). The biggest advantage of this approach is that it is based on the most frequent cluster combination, which provides the opportunity to obtain stimuli that resemble real word letter combinations in syllables, and it also includes the calculation of the orthographic neighborhood (e.g., Coltheart’s N). However, in this case as well, the generated pseudowords do not have specific grammatical class markers but are merely clusters of word-like syllables. In addition, these programs usually already contain a list of words from which pseudowords are generated. Consequently, it is only possible to create stimuli with a fixed input embedded in the particular tool. Another problem a researcher may encounter is that these tools are only developed for few languages such as English (MCWord and WordGen), French (WordGen), and Dutch (WordGen), and due to the noneditability of their functions, it is not possible to obtain stimuli in other languages.

One of the recent breakthrough-advancements in pseudoword production is the Wuggy algorithm (Keuleers & Brysbaert, 2010), which can be used for the construction of polysyllabic pseudowords that obey phonotactic constraints. The Wuggy algorithm efficiently produces legal sequences (words and pseudowords) in the language of choice using bigram chains that are built based on language specific lexicons (Keuleers & Brysbaert, 2010). This approach alleviates the combinatorial explosion problem that is present in solutions that are based on production of all combinations of subsyllabic elements (i.e., onset, nucleus and coda) that are legal in the language of choice (Keuleers & Brysbaert, 2010). The Wuggy algorithm accounts for the probability of the occurrence of subsyllabic elements by matching the language pattern with the source word used as a reference (Keuleers & Brysbaert, 2010). The Wuggy algorithm can be downloaded and partially customized. The words from which the output will be generated can be typed independently or imported as a list, allowing more flexibility in the selection of real words. These can be, for example, words with specific emotional valence. The algorithm’s functions allow researchers to determine the length of pseudowords with respect to real words by matching subsyllabic segment length, which results in pseudowords that have the same syllable structure as the input words. Wuggy also calculates orthographic neighborhoods using the OLD20 method, which quantifies the similarity of the given pseudoword generated to the 20 most similar words from the corpus. With the function of splitting the word into syllables before generating the output and adjusting the values of the subsyllabic elements, it is possible to generate pseudowords from the input coming from English (Tucker & Brenner, 2017), Dutch (Heyman et al., 2015), German (Hasenäcker et al., 2017), Turkish (Erten, 2013), Basque (Ferré et al., 2015), French (De Simone et al., 2021), Spanish (Aguasvivas et al., 2018), and Bulgarian (Shtereva et al., 2020). This makes Wuggy a tool with the most extensive and detailed options that can be customized for not only positional languages but also those with more complex morphology, such as Polish. Given that Wuggy can compute the psycholinguistically relevant properties of words, such as OLD20, that play an important role in pseudoword construction, we used this tool to produce Polish pseudowords. However, by default, the Wuggy algorithm does not allow for constructing stimuli with the predefined features, such as suffixes indicative of pseudowords belonging to a grammatical class. To address this gap, we provide a pipeline that employs the Wuggy algorithm and facilitates the production of pseudowords of a particular grammatical class in inflectional languages with additional fine control over other significant psycholinguistic features of the output pseudowords.

## Pipeline for Polish Pseudonoun and Pseudoverb Generation and Initial Selection

**Fig. 1:**
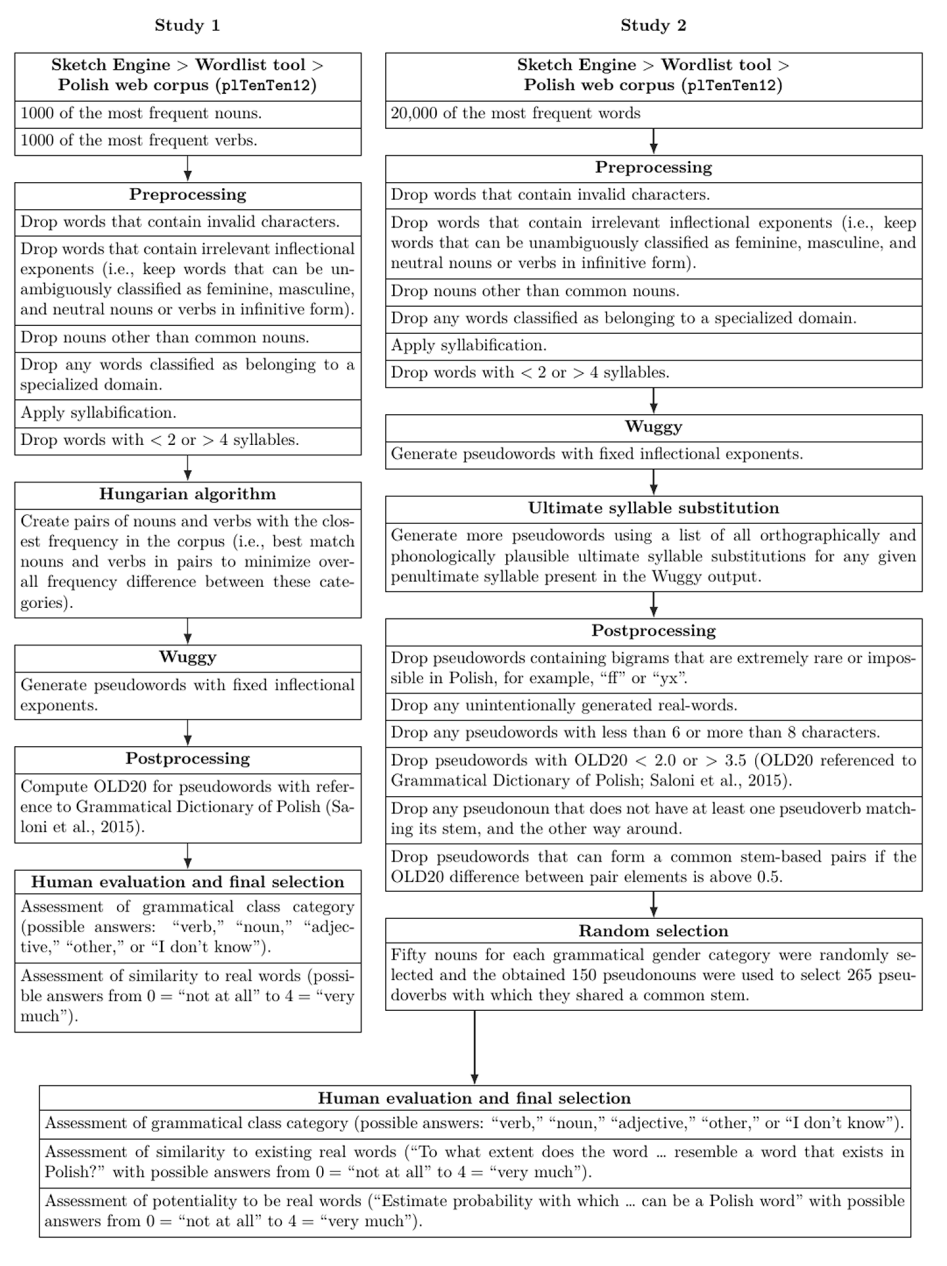
Schematic depiction of pseudoword generation pipelines used in Study 1 and 2. Further implementation details are provided in the main text and Python code is provided in the SOM

We used Python as the primary scripting language to generate Polish pseudowords. The process of pseudoword generation is presented in Fig. 1 and outlined in more detail in subsections “Stimuli Generation and Selection” for Studies 1 and 2. We conducted two studies that allowed us to evaluate the stimuli using ratings from online surveys for crosscheck of machine and human stimuli evaluation. This resulted in two sets of pseudowords containing information on their grammatical properties (e.g., grammatical class, grammatical gender of pseudonouns) and psycholinguistically relevant properties (e.g., number of letters, number of syllables, and OLD20). To resolve the problem of the probability of letter-cluster occurrences in the beginning of the word (Hawelka et al., 2013), we also provided the first-syllable frequency for each pseudoword that we extracted from the Polish corpus (Lexical Computing CZ s.r.o., 2015; Jakubíček et al., 2013; Suchomel & Pomikálek 2012). The supplementary materials also contain results of a rating study (e.g., the percent of correct identifications of grammatical class, mean and standard deviation of ratings of similarity to a real word).

To select the most characteristic grammatical endings for each of the chosen grammatical classes, we used data from the most common Polish language dictionaries, which contain summaries of morphological features embedded in nouns and verbs (Drabik et al., 2018; Dunaj, 2012; Sobol 2002). We extracted specific exponents for feminine, masculine, and neuter nouns in their nominative form and verbs in the infinitive form for Stimuli Set 1 and kept the last syllable of the real word in Stimuli Set 2.

Importantly, the proposed method of pseudoword stimuli generation can be extended to any other grammatical category and, thus, offers a broader framework on pseudoword construction for all languages that rely on words’ morphological features to signify their grammatical properties. This capability also easily extends beyond grammatical categories to all grammatical, syntactic, or other features that go beyond the semantics and are indicated by word’s morphology in a given language (e.g., gender in nouns and tense in verbs). Researchers should verify that such an indication is unambiguous for the language in which they plan to conduct research. For example, in Polish, inflection of a verb can correspond to the gender of the agent in the sentence, and feminine nouns and several masculine nouns use the same suffixes - ‘*Koleżanka(f.)/Kolega(m.) otwieradrzwi*.’ (‘*A colleague opens the door*’). Thus, pseudowords pertaining to any of these categories would not be unambiguously distinguishable. For that reason, we abstained from the generation of this type of stimuli in our example pseudoword sets, and we used only infinitive verbs and excluded masculine nouns that have suffixes identical to feminine nouns. The scripts used to generate the two initial sets of pseudowords for this paper are available as supplemental online material (SOM) at https://gin.g-node.org/SocGramLab/pseudo-word-stimuli-with-arbitrary-constrains.git.

### Study 1: Pseudowords with Different stems

#### Stimuli Generation and Selection

We extracted the most frequently used Polish words using the Sketch Engine (https://app.sketchengine.eu) framework (Kilgarriff et al., 2004). Specifically, we used the Wordlist tool together with the Polish web corpus (Lexical Computing CZ s.r.o., 2015; Jakubíček et al., 2013; Suchomel & Pomikálek 2012). This corpus comprises more than 7 billion words in 22 million online documents that were crawled in June 2012 to obtain over 36 million unique word forms. As this was the primary data input for Study 1, we selected 1,000 of the most frequent verbs and 1,000 of the most frequent nouns.

The initial word lists were filtered using the following procedure. First, we removed duplicate entries and word forms that contained any characters not in the standard Polish alphabet (i.e., word forms that were included in the corpus due to errors and defective procedures, such as optical character recognition). Next, to obtain additional characteristics of each word, we used the Grammatical Dictionary of Polish (Saloni et al., 2015), which includes a Morfeusz 2 inflectional analyzer and generator (Kieraś & Woliński, 2017). For verbs, we selected only infinitives. For nouns, only the nominative forms of singular masculine, feminine, and neuter forms were preserved (further classification details, including lists of suffixes used to assign these categories, are available in the SOM). Furthermore, we kept only nouns classified as common nouns and disregarded any words that were classified as belonging to a specialized domain, such as music (Latin-based, e.g., *allegro*, or “fast music tempo”), linguistics (relating to metalinguistics, e.g., *przypadek*, or “case”), or archaic (rarely used in modern Polish, e.g., *dziewierz*, or “brother-in-law”).

Next, words were hyphenated according to Polish syllabification rules using Pyphen (Kozea Community, 2018). We set the language dictionary to “pl_PL” and the minimum number of characters of the first and last syllable to 1. We eliminated words that had less than two syllables to ensure that words clearly indicated the grammatical class with a suffix in the last syllable. We also eliminated words that had more than four syllables to avoid obtaining pseudowords with extremely different lengths.

To ensure that the input nouns and verbs were matched with respect to their usage frequency in real language, we applied the Hungarian algorithm to create pairs of nouns and verbs with the closest frequency in the corpus (Kuhn, 1955). In short, for a given 2D array containing some weights (interpreted as the penalties or costs of assigning items that typically belong to two distinct categories to pairs), this algorithm solves the assignment problem in which the sum of weights (i.e., total penalty/cost) is minimized. In our case, the array represented the difference between the frequencies of verbs and nouns in the corpus. This resulted in a collection of source nouns and verbs with the most closely matched frequencies. The average absolute frequency difference for all pairs, normalized by the average frequency of the items in the pair, was 0.08 with a standard deviation of 0.27 (p.d.u. based on the difference between words with respect to their absolute frequency, which is a Sketch Engine metric defined as the direct count of how many times a given item was found in the corpus).

Next, the word pairs were inputted into the Wuggy program (Keuleers & Brysbaert, 2010) with the Polish module, which generated pseudowords based on the provided real words without altering the suffix that denotes grammatical class. In order to preserve the suffix of the original source word we used Wuggy’s Matching Expression feature that provides a way to require pseudowords to match provided regular expression (Keuleers & Brysbaert, 2010; further information about regular expressions is widely available online, for a technical description refer to IEEE et al., 2018). For example, using the source word “verbifying” and the regular expression “.+ing$” would result in Wuggy generating only pseudowords based on the source word and ending in –ing. In our pipeline, for each real word used as a source of pseudowords we used a regular expression that required generated pseudowords to retain the ultimate syllable from the source word. For the obtained pseudonouns and pseudoverbs, we calculated the frequencies of their initial syllables (all but the last one), and we verified that these two groups of stimuli were similar in terms of a syllable frequency distribution (Kolmogorov-Smirnov test was insignificant, suggesting that the two independent samples are drawn from the same distribution). We repeated this test for the first syllables only, and this test was also insignificant, suggesting that our pseudonouns and pseudoverbs did not differ with respect to the frequency of their first syllables. We obtained a total of 640 stimuli (320 pseudonouns, e.g., *syjcol*, *setylda*, and 320 pseudoverbs e.g., *chlocić*, *osordać*), each of which contains six to eight letters. For these pseudowords we computed OLD20 values with reference to the Grammatical Dictionary of Polish (Saloni et al., 2015).

#### Stimuli Evaluation Procedure

The goal of this procedure was to test the pseudowords with multiple stems in terms of association of OLD20 values, based on people’s pseudowords perception, and extraction of grammatical class clearly indicative of a pseudoword being a verb or a noun. Participants were invited to take part in an online study and were randomly assigned to one of 16 lists that presented 40 pseudowords in a fixed random order. Before the stimuli evaluation task, participants were asked to provide basic demographic information, such as age, gender, education, foreign language knowledge, and diagnosed language processing disorders, such as dyslexia or other language impairments. We also asked whether Polish was their native language.

For each pseudoword, we asked two questions. The first concerned the similarity of the presented pseudoword to any real word in Polish (e.g., “To what extent does the word *poceda* resemble a word that exists in Polish?”), with answers ranging from 0 (not at all) to 4 (very much). The second question concerned the grammatical class of the presented pseudoword (e.g., “Which grammatical class might the word *poceda* be?”). Participants had to select one of the presented options: “verb,” “noun,” “adjective,” “other,” or “I don’t know.”

#### Participants

A total of 328 people participated in this study (247 women, 76 men, 4 people who refused to indicate their gender, and 1 person who declared their gender to be “other”; *M*_*age*_ = 31.06 years, *SD*_*age*_ = 8.58 years). Twenty-four people were excluded from the data analysis due to their declaration of diagnosed language processing disorders, and one person was excluded because their declared native language was not Polish. We based the final evaluation of pseudowords in this study on ratings from 303 people.

#### Resulting Word List for Study 1

In order to choose stimuli that were most clearly perceived as verbs or nouns, we applied the following criteria. First, the accuracy of grammatical class identification had to be above 80% to ensure that words were commonly recognized as either verbs or nouns. Moreover, we only chose pseudowords that were rated significantly less than 3 on the scale measuring similarity to Polish words to ensure that the pseudowords were not too similar to any existing word. The final dataset of pseudowords contained 26 feminine, 79 masculine, and 12 neuter nouns as well as 41 verbs in the infinitive form. A summary of the basic properties of the words is presented in Table 1, and the dataset containing all stimuli and those selected for our list is available in the SOM.

**Table 1:** *Properties of Study 1 dataset*

Variables	Pseudonouns	Pseudoverbs
	M (SD)	M (SD)
Similarity to real words	1.93 (0.35)	2.19 (0.27)
Correct identification (%)	90.72 (6.24)	93.86 (6.51)
Orthographic neighbors (OLD20)	2.59 (0.18)	2.60 (0.15)

The results of a Spearman correlation indicated a nonsignificant relationship between OLD20 values and human average ratings of pseudoword similarity to real words for pseudonouns (*r*(115) = 0.12, *p* = .21) and a nonsignificant relationship between OLD20 values and human ratings for pseudoverbs (*r*(39) = − 0.23, *p* = .14). These findings indicate that the OLD20 measure of similarity to existing words and human subjects’ judgment of pseudowords’ similarity to real words are independent. Furthermore, even though the two correlations were not significant, the correlation of pseudonouns and OLD20 values was positive and the correlation of pseudoverbs and OLD20 values was negative. Therefore, we applied using the Fisher’s Z-Transformation to compare whether there was a significant difference between the two correlation coefficients. Importantly, the comparison indicated that the two correlation coefficients were similar *z* = -1.89, *p* = .06. Therefore, we can conclude that the pseudonouns and pseudoverbs are similarly unrelated to OLD20 values.

### Study 2: Pseudowords with Shared Stem

#### Stimuli Generation and Selection

We extracted the most frequently used Polish words using the Sketch Engine (https://app.sketchengine.eu) framework (Kilgarriff et al., 2004). Specifically, similarly to Study 1, we used the Wordlist tool together with the Polish web corpus (Lexical Computing CZ s.r.o., 2015; Jakubíček et al., 2013; Suchomel & Pomikálek 2012), however, for Study 2 we selected 20,000 of the most frequent words as the primary data input (initial corpus).

Our aim in this study was to obtain pairs that consist of pseudoverbs and pseudonouns that share a common stem but differ with respect to their suffixes (which enable distinction between grammatical classes). To achieve this goal we developed a procedure for plausible last-syllables substitution that resulted in the generation of pseudoword pairs with a common stem. The initial steps of the procedure employed in this study were analogous to the steps used in the first study. However, here, we did not use the Hungarian algorithm to select verb–noun pairs of closest corpora frequency but instead utilized (as an input to the Wuggy program) all nouns and verbs that were identified in the initial corpus and meet same inclusion criteria as in Study 1. Similarly to Study 1, we only kept words containing standard Polish alphabet characters—verbs in the infinitive form and common nouns in singular nominative masculine, feminine, and neuter form. Next, based on the pseudowords generated by Wuggy, for each penultimate syllable, we constructed a list of all ultimate syllables that could follow it. To that end we constructed a list of penultimate syllables that were present in pseudowords generated by Wuggy. For each penultimate syllable we produced a list of all plausible ultimate syllables that can follow it according to the Wuggy output. This resulted in an exhaustive list of all orthographically and phonologically plausible ultimate syllable substitutions conditioned on the penultimate syllable present in the Wuggy output. All ultimate syllables of the obtained pseudowords were possible inflectional noun/verb endings because, as in Study 1, the Wuggy was configured to retain the last syllable of the pseudowords generated. Each ultimate syllable clearly indicated the grammatical class of pseudoword that it terminated. Based on these lists, more pseudowords were generated by means of last-syllable substitution (all possible substitutions were used conditioned only on the penultimate syllable). This approach minimized the probability of an accidental introduction of impossible or very rare suffixes to the generated pseudowords. Since we aimed for the production of pseudoverb–pseudonoun pairs, for any further processing, only pseudowords with stems that had been assigned with (jointly) at least one noun-indicating and at least one verb-indicating suffix were considered (we removed pseudowords with stem that was present in only one grammatical category). To further ensure orthographic and phonological plausibility of the pseudowords, we removed words that contained bigrams that are extremely rare or impossible in Polish, for example, “ff” or “yx” (for the full list, please refer to the SOM). Using the Grammatical Dictionary of Polish (Saloni et al., 2015), we also removed any real words that could have resulted from the syllable substitution procedure. Furthermore, we only kept words containing 6–8 characters. For the obtained pseudowords the OLD20 metric was computed with reference to the Grammatical Dictionary of Polish (Saloni et al., 2015), and to ensure that the resulting pseudowords were orthographically and phonologically plausible but not too similar to real words, we removed any pseudowords that had OLD20 below 2.0 or above 3.5. Additionally, because we intended to produce pairs of stimuli that contained items of comparable properties, after the above filtering based on OLD20, we only kept the pseudonoun and pseudoverb sets that shared a common stem. In other words, a pseudonoun was removed from the set if no pseudoverb shared a stem with it, and the other way around. For the same reason we only kept pseudonouns and pseudoverbs that were able to form common stem-based pairs for which the difference in OLD20 measure computed with reference to the Grammatical Dictionary of Polish (Saloni et al., 2015) between potential pair elements was less than 0.5 and contained the same number of characters within potential pair items. Furthermore, based on the noun suffixes, we assigned grammatical genders to the obtained pseudonouns and only kept words for which their suffixes allowed for unambiguous grammatical gender classification. At this stage, we obtained 385 pseudonouns (121 masculine, 203 feminine, and 61 neuter). Next, we randomly selected 50 nouns for each grammatical gender category. The obtained 150 pseudonouns were used to select 265 pseudoverbs with which they shared a common stem. Altogether, these constituted a set of 415 pseudowords (e.g., *grocunek, grocukać*, *rocezja, rocerać*) that were evaluated by human subjects. Some pseudoverbs shared stems with more than one pseudonoun (e.g., *mocejać, mocetła, mocezja*), and some pseudonouns shared stems with more than one pseudoverb (e.g., *rorekia, rorerać, roregać*).

#### Stimuli Evaluation Procedure

The goal of this procedure, analogous to that in Study 1, was to test the stimuli in terms of association of OLD20 values with people’s pseudoword perceptions and extraction of grammatical class clearly indicative of a pseudoword being a pseudoverb or pseudonoun. In this study, however, we used stimuli that shared a stem to more strongly emphasize grammatical class differences in the last syllable. Participants were invited to take part in an online study and were randomly assigned to one of 12 lists that presented 35 pseudowords in a fixed random order. As in Study 1, before the stimuli evaluation task, participants were asked to provide basic demographic information, including age, gender, education, foreign language knowledge, and diagnosed language processing disorders, such as dyslexia or other language impairments. We also asked whether Polish was their native language. Three questions were asked for each pseudoword. The first concerned the possibility that a word could be a real Polish word (i.e., whether a pseudoword was constructed in line with Polish lexical rules; “Estimate probability in which *poceda* can be a Polish word”). The second question asked about the similarity of the presented pseudoword to any real Polish word (e.g., “To what extent does the word *poceda* resemble a word that exists in Polish?”). For both of these questions, answers ranged from 0 (not at all) to 4 (very much). The third question concerned the grammatical class of the presented pseudoword (e.g., “Which grammatical class might the word *poceda* be?”). Participants had to select one of the presented options: “verb,” “noun,” “adjective,” “other,” or “I don’t know.” Finally, we asked how carefully the participants filled out the questionnaire, with answers ranging from 0 (careless) to 3 (very carefully).

#### Participants

A total of 312 people participated in this study (228 women, 80 men, 2 people who refused to indicate their gender, and 2 people who declared their gender to be “other”; *M*_*age*_ = 31.5 years, *SD*_*age*_ = 9.51 years). We excluded 22 people from the data analysis due to their declaration of diagnosed language processing disorders and four people based on their self-evaluated low attention (0, or careless). We thus based our evaluation of the pseudowords with different stems on the ratings of 286 people.

#### Resulting Word List for Study 2

In order to select stimuli, we applied the following criteria. First, as in Study 1, the accuracy of grammatical class identification had to be above 80% to ensure that words were commonly recognized as either pseudoverbs or pseudonouns. Moreover, we only chose pseudowords that were considered possible in Polish (ratings significantly higher than 3), as this indicated phonological acceptance. Furthermore, we only chose words that were rated as significantly less than 3 on the scale measuring similarity to Polish words, as this ensured that the pseudowords were not too similar to any existing word. In addition, the OLD20 measure (*M* = 2.81 for pseudonouns and *M* = 2.84 for pseudoverbs) was used to select pseudowords that were not too similar to or too distant from the real words on which they were based and to construct pseudoword noun–verb pairs with similar OLD20 values. Next, using the Hungarian algorithm, we paired pseudowords to establish noun–verb pairs with a shared stem and the most similar properties. This resulted in a final dataset of pseudowords containing 121 pseudonoun and pseudoverb pairs constructed from 93 pseudoverbs and 47 pseudonouns. Pairs were not exclusive with respect to stem (one pseudoverb could share a stem with more than one pseudonoun and one pseudonoun could share stem with more than one pseudoverb). A summary of the basic properties of the pseudowords set is presented in Table 2, and the dataset, including all stimuli and those selected for our list, is available in the SOM.

The Spearman correlation between the similarity of pseudowords to real Polish words and the OLD20 measure was significant for pseudoverbs (*r*(91) = − 0.31, *p* < .05) and nonsignificant for pseudonouns (*r*(45) = − 0.23, *p* = .13). In the case of pseduoverbs, we obtained a significant result of a negative low correlation, which means the higher resemblance to a real Polish word, the lower the OLD20 value. It has to be noted, however, that the sample size for pseudoverbs was higher than in case of pseudonouns, likely driving the significance of the correlation coefficient. When we compared the two correlation coefficients using a comparison of correlation from independent samples using Fisher’s Z-Transformation method, the result indicated a nonsignificant difference: *z* = − 0.47, *p* = .64. This suggests that pseudonouns and pseudoverbs are similarly unrelated to OLD20 (as in Study 1) and that the significance of the correlation between pseudoverbs and OLD20 is likely an artifact.

The Spearman correlation between OLD20 values and the possibility that the presented pseudoword is a Polish word were calculated separately for the pseudonoun and pseudoverb in each pair. The results indicated a nonsignificant relationship between OLD20 values and the possibility that the presented pseudoword could be a Polish word for both pseudoverbs (*r*(91) = − 0.10, *p* = .36) and for pseudonouns (*r*(45) = − 0.11, *p* = .46).

Additionally, the z-score of comparison between the correlation coefficients for pseudoverbs and pseudonouns in relation to OLD20 indicated that the two correlation coefficients were similar: z = 0.05, *p* = .96.

**Table 2:** *Properties of Study 2 dataset*

Variables	Pseudonouns	Pseudoverbs
	M (SD)	M (SD)
Similarity to real words	1.40 (0.48)	1.39 (0.39)
Possibility of being a real word	1.69 (0.49)	1.66 (0.34)
Correct identification (%)	90.69 (5.45)	91.38 (5.10)
Orthographic neighbors (OLD20)	2.82 (0.35)	2.83 (0.32)

## General Discussion

In this paper, we have presented a pipeline for pseudoword generation and two sets of pseudoverbs and pseudonouns that can be used in language research. Especially we focused on example applications in languages that use specific suffixes from parts of speech. We also demonstrated how measures of orthographic distance (e.g. OLD20) between the generated pseudowords and real words can be used to obtain pseudowords with varying degree of similarity to real words. By providing an open script, we create a possibility for researchers to use it to produce pseudowords mainly in inflectional languages, in which the value of a grammatical class is determined by the exponent located at the end of a word. As the preparation of stimuli for psycho- and neurolinguistic research requires careful consideration, we proposed a detailed analysis of specific language properties, such as real word frequency, phonological congruence, first-syllable frequency, orthographic distance to nearest real words (OLD20), and grammatical class adequacy in Polish. In addition to controlling the frequency of the real words used to create the pseudowords and the OLD20 measure, we considered it necessary to also obtain human ratings for pseudoword evaluation. Instead of using zero-one rating systems, such as “word” and “nonword,” as in the English Lexicon Project lexical decision task (Balota et al., 2007), we used a 5-point scale, which allowed us to achieve greater specificity in evaluating the prepared stimuli.

Overall results of our studies indicate that there is no systematic relation between average human ratings considering similarity to existing Polish words and OLD20. In Study 1 the correlation was nonsignificant for pseudonouns and for pseudoverbs and the two correlation coefficients were of similar magnitude. In Study 2, the correlation of pseudonouns and OLD20 was also nonsignificant, but we observed a significant correlation of pseudoverbs and OLD20. One possible explanation for the discrepancies between human and computerized evaluation of pseudowords is the effect of possible associations and interpretations that a pseudoword may trigger in human subjects that are not captured by computerized assessment. We however, treat this one significant result with caution because the sample size of pseudoverbs was twice as large as the sample of pseudonouns, likely driving the significance of the correlation coefficient, and the comparison of the two correlation coefficients was not significant. Furthermore, in terms of rating the possibility to be a real Polish word, both pseudoverbs and pseudonouns were similarly not correlated to OLD20 values. Overall, the pattern of results suggest that the presented pseudoverbs and pseudonouns are of similar quality. Additionally, in Study 2 we found similar negative trends for correlation coefficients of OLD20 and both question responses for each of pseudowords’ grammatical categories. This indicates, that both for pseudonouns and verbs, the higher the OLD20 value the lower the participants’ rating of pseudowords’ similarity to real Polish words and possibility to be perceived as a potential Polish word. In other words, the less LD-operations (letter substitution, deletion or insertion) are required to transform a real word into a pseudoword, the more pseudoword is judged as resembling a real word. This result is in line with previous research that has investigated the impact of the OLD20 measure and lemma frequency on the results of lexical decision and word-naming task performance (Kresse et al., 2012). In the presented task low-OLD20 scores of pseudowords elicited higher naming error rates but shorter reaction time than words with high-OLD20 scores. Thus, the less distant a pseudoword to real word is, the more fluently it can be perceived as a natural language unit, but at the same time, the opportunity for naming error becomes greater. Bearing in mind the research of Kresse et al., (2012) where OLD20 showed impact on task performance, pseudowords with average OLD20 in the attached sets of pseudonouns and pseudoverbs allow to exclude OLD20 impact on perception while measuring e.g. reaction times across the grammatical categories. A final conclusion regarding the two presented datasets is that even when using highly reliable tools for pseudoword construction, such as Wuggy, it is useful to test how the words are perceived by potential participants.

The presented approach allowed us to produce and share a unique set of stimuli to study the impact of grammatical classes in psycholinguistic studies of language processing for testing grammatical processing differences between verbs and nouns. We used them as examples of the application of a stimulus-producing algorithm to linguistic research. The stimuli sets we created can be used by other researchers. The first set is more suitable for general pseudowords related to linguistic research, as it provides a variety of pseudowords with different stems. This means it can be used in research regarding grammatical classes but also that its items can be used as fillers in other psycholinguistic studies. The second set contains pseudowords that differ only in grammatical class exponent and, thus, provides an opportunity to unify other pseudoword properties between pseudonouns and pseudoverbs. This makes it more appropriate for specific grammatical class-related research.

To conclude, pseudowords provide a convenient tool for studying the morphosyntactic properties of language processing because of the unique features they can be equipped with or dislodged from. As artificial language units, they might be a good source of knowledge regarding language processing and its properties related to human behavior. They can be used as stimuli for investigating language properties beyond semantics, such as iconicity of sound (Köhler, 1929; Sapir, 1929) or morphological carriers of linguistic information that have an impact on real-world feature perceptions (Adelman et al., 2018; Formanowicz et al., 2017). However, pseudowords may also have drawbacks that researchers should be aware of that may bias their results, for example, frequency of the first syllable (Carreiras & Perea, 2004), phonotactic violations, or the influence of the first letter (Scaltritti et al., 2018) on the perception of the pseudoword. Because pseudowords occupy a gray area between nonwords and real words, the process of fine tuning their properties is a challenging multifaceted problem. However, pseudowords are immensely informative for the psychology of language.
